# Using mHealth to improve eye care in remote areas of Iran

**Published:** 2019-12-17

**Authors:** Marzieh Katibeh, Batool Mousavi, Masomeh Kalantarion, Hamideh Sabbaghi, Ehsan Abdolahi, Homayoun Nikkhah, Hamid Ahmadieh, Per Kallestrup

**Affiliations:** 1 Centre for Global Health, Department of Public Health, Aarhus University, Aarhus, Denmark; 2 Department of eHealth, Asre Danesh Afzar (ADA), Tehran, Iran.; 3 World Health Organization Collaborating Centre for Eye Health and Prevention of Blindness, Ophthalmic Research Centre, Shahid Behesthi University of Medical Sciences, Tehran, Iran.; 4 World Health Organization Collaborating Centre for Eye Health and Prevention of Blindness, Ophthalmic Research Centre, Shahid Behesthi University of Medical Sciences, Tehran, Iran.; 5 Department of eHealth, Asre Danesh Afzar (ADA), Tehran, Iran.; 6 World Health Organization Collaborating Centre for Eye Health and Prevention of Blindness, Ophthalmic Research Centre, Shahid Behesthi University of Medical Sciences, Tehran, Iran.; 7 World Health Organization Collaborating Centre for Eye Health and Prevention of Blindness, Ophthalmic Research Centre, Shahid Behesthi University of Medical Sciences, Tehran, Iran.; 8 Centre for Global Health, Department of Public Health, Aarhus University, Aarhus, Denmark.

**Primary health workers are using a new digital mHealth tool to support eye health screening and management in Iran.**

**Figure:**
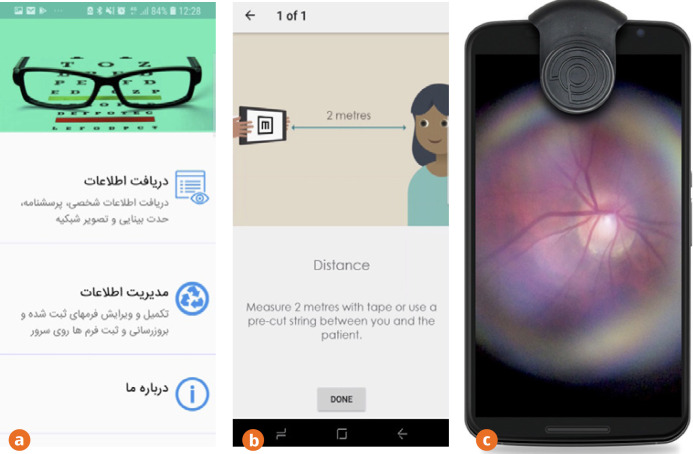
Primary health care workers use the three integrated apps in turn to: a) capture demographic data and needs assessment, b) measure visual acuity using Peek Acuity and c) take a photograph of the retina using Peek Retina.

Mobile communication technology in health (mHealth) offers opportunities to improve prevention and care for non-communicable diseases (NCDs).^1^ Most evidence comes from high-income countries;^2^ however, recent studies show that this technology is also effective at community level in low- and middle-income countries (LMICs).^3^,^4^ It is possible to enhance health care through mobile technology, both on- and offline, in different settings.^5^

In Iran there is one ophthalmologist and one optometrist per 40,000 and 45,000 people, respectively. Although this meets the World Health Organization's recommendations, the distribution in the country is uneven.^6^ Recent population-based studies showed that the proportion of avoidable eye disorders is high even in areas with available human resources and infrastructure due to inadequate prevention.^7^,^8^

mHealth, also known as telemedicine, may facilitate cost-effective use of available resources and decrease unnecessary workload on referral centres. To enable early detection and better management and monitoring of vision-threatening conditions, provide timely follow-up and prevent ocular morbidity, we designed a community-based mHealth screening, management and monitoring tool to be integrated into the national health care system. With this tool, primary health care (PHC) workers – the community health providers known as Behvarz in Iran – have the opportunity to screen the population in remote areas, upload data and digitally consult with an ophthalmologist in an urban referral centre.

## How does it work?

The tool has two components: a mobile application and web-based software. The mobile application consists of digital survey forms, an integrated software application (Peek Acuity) for obtaining visual acuity (VA),^9^ integrated hardware for optimizing retinal imaging (Peek Retina),^10^ and a management system for the PHC workers.

The forms and screening tests were developed based on focus group discussions with professionals and health providers.^11^

There are four steps:

1. The PHC workers use the mobile application to enter each patient's demographic and eye health history data, visual acuity and retinal images.
2. The information is uploaded to the customised web-based system.
3. An ophthalmologist in a reading centre reviews the information on the web-based system and provides a management plan.
4. Participants receive their results and follow-up advice, if needed, using an automated SMS (text message) system.

## Testing the mHealth tool

An intervention trial on community members was carried out to test the effectiveness and acceptability of the tool. A representative sample of 50+ residents from 27 enumeration areas were enrolled through door-to-door visits of the households with a random and compact segment sampling method. Ethical approval was granted by Shahid Behesthi University of Medical Sciences in Tehran.

The study had three arms:

1. mHealth intervention: using mobile application for data collection and screening tests.
2. Conventional intervention: using paper-based forms, Snellen chart for measuring VA and fundus photography equipment for fundus imaging.
3. Control arm: using paper-based forms, no further intervention.

Participants with presenting VA≤20/40 in either eye were referred to an ophthalmologist for further evaluation. An ophthalmic assistant conducted retinal imaging, using Peek Retina, at the local PHC units. Retina specialists in a central reading centre reviewed retinal images and, if necessary, made further referral plans.

### Results

Over 3,000 residents were enrolled, with 92.1% in the control, 78.4% in the mHealth and 57.7% in the conventional group agreeing to participate.

Of 1,508 participants in vision screening (mHealth = 873), (conventional = 635), 608 (40.3%, 95%CI: 37.8–42.8) had ≤20/40 vision in at least one eye and were therefore referred for evaluation (referral rate 35.9% in conventional vs. 43.5% in the mHealth arm, not significant after adjusting for age and need for eye care.

Fundus evaluation was performed in 756 participants with >20/40 presenting visual acuity. Of the 756, 45 (0.6%) had poor quality images (20 [0.5%] in conventional and 25 [0.75%] mHealth arms). Of 711 people with fundus evaluation, 173 (24.3%) had abnormal images (79 [19.6%] in conventional and 94 [26.9%] mHealth arms).

### Implications

The findings show a high level of eye care need in the study population. PHC workers were able to examine the eye and transmit images for expert assessment using the mHealth tool.

Making a definite diagnosis and grading of disorders were beyond the objectives of the study; nevertheless, in those who were referred based on fundus imaging, the following abnormalities were found: diabetic retinopathy, age-related macular degeneration, glaucoma, high myopia, chorio-retinal scar, myopic fundus with chorio-retinal atrophy, retinal scars and hyper-pigmentation.

Geographic location and socioeconomic status are factors that may inhibit service provision, use and continuity of care. Appropriate mHealth solutions may provide access to specialist consultation services and early interventions. This mHealth eye-care approach delivers health care to populations that have limited access to specialist services and improves the health care where there are only partial services.

**Figure:**
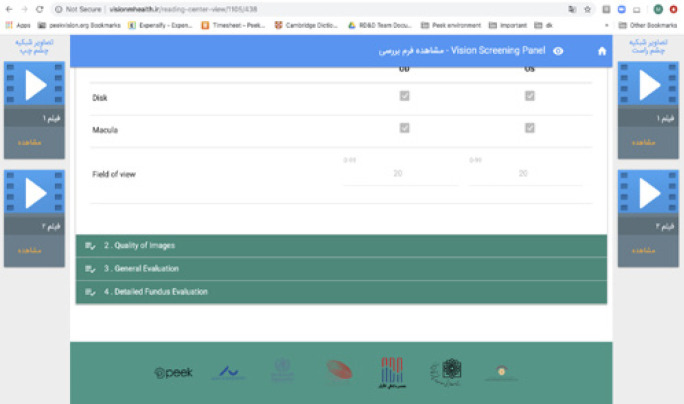
Ophthalmologist receive information through the online system and evaluate patients' records. The screening results and follow-up advice, if needed, are sent to patients via SMS (text message).

Many advantages of this mHealth solution were identified, including improved resource use, early intervention, avoidance of unnecessary transportation, community-based delivery and engagement, combined medical education and research, cost efficiency, improved medical record documentation and an increased coverage of care.

The mHealth tool is being updated following feedback from different users.

**Acknowledgement:** The authors would like to express their sincere gratitude to the Peek Vision team in the UK for their great contribution to developing the mHealth tool in this project. We also express our sincere gratitude to Ardeshir Montaseri, the CEO, and the IT team at the Asre Danesh Afzar Co. who adopted and managed this mHealth tool.
